# Ambiguity-Free Optical–Inertial Tracking for Augmented Reality Headsets

**DOI:** 10.3390/s20051444

**Published:** 2020-03-06

**Authors:** Fabrizio Cutolo, Virginia Mamone, Nicola Carbonaro, Vincenzo Ferrari, Alessandro Tognetti

**Affiliations:** Information Engineering Department, University of Pisa, Via G. Caruso 16, 56122 Pisa, Italy; virginia.mamone@endocas.unipi.it (V.M.); nicola.carbonaro@unipi.it (N.C.); vincenzo.ferrari@unipi.it (V.F.); alessandro.tognetti@unipi.it (A.T.)

**Keywords:** augmented reality, optical tracking, computer vision, perspective 3-point problem, inertial tracking, hand–eye calibration, sensor fusion, Kalman filter, head-mounted display

## Abstract

The increasing capability of computing power and mobile graphics has made possible the release of self-contained augmented reality (AR) headsets featuring efficient head-anchored tracking solutions. Ego motion estimation based on well-established infrared tracking of markers ensures sufficient accuracy and robustness. Unfortunately, wearable visible-light stereo cameras with short baseline and operating under uncontrolled lighting conditions suffer from tracking failures and ambiguities in pose estimation. To improve the accuracy of optical self-tracking and its resiliency to marker occlusions, degraded camera calibrations, and inconsistent lighting, in this work we propose a sensor fusion approach based on Kalman filtering that integrates optical tracking data with inertial tracking data when computing motion correlation. In order to measure improvements in AR overlay accuracy, experiments are performed with a custom-made AR headset designed for supporting complex manual tasks performed under direct vision. Experimental results show that the proposed solution improves the head-mounted display (HMD) tracking accuracy by one third and improves the robustness by also capturing the orientation of the target scene when some of the markers are occluded and when the optical tracking yields unstable and/or ambiguous results due to the limitations of using head-anchored stereo tracking cameras under uncontrollable lighting conditions.

## 1. Introduction

The primary goal of visual augmented reality (AR) technology is to enrich the visual perception of the surrounding space by overlaying three-dimensional (3D) computer-generated elements on it in a spatially realistic manner. In any AR application, the problem of correctly defining the spatial location of the digital elements with respect to the real scene is the principal factor that provides the user with a sense of perceptive congruity [1,2]. In order to satisfy the locational realism of the AR view and achieve an accurate spatial alignment between real-world scene and virtual elements, the process of image formation of the virtual content must be the same of the real-world scene [3]. To this end, the conditions to be satisfied are twofold. First, the intrinsic of the virtual viewpoint (i.e., the rendering camera) must be properly determined through a calibration routine (e.g., [4]). Secondly, the online estimation of the pose of the target scene in relation to the stereo cameras dictates the proper placement of the virtual objects in the AR scene (i.e., the extrinsic parameters). This task is typically accomplished by means of a tracking device that provides in real time the pose of the target scene to be augmented with respect to the real viewpoint. The real viewpoint corresponds to one or two display-anchored camera(s) in video see-through (VST) displays, and to the user’s eye(s) in optical see-through (OST) displays.

Most AR systems rely on optical tracking methods to estimate the 6-degrees-of-freedom (DoF) pose: 3 DoF for the position (x, y, z) and 3 DoF for the orientation (roll, pitch, yaw). Optical tracking can be grouped into two main classes: markerless methods, and marker-based methods.

Markerless tracking techniques that require little or no a-priori knowledge of the environment generally rely on SLAM (Simultaneous Localization And Mapping) algorithms to estimate the pose of the target scene. Among such methods, extensible tracking techniques attempt to integrate and refine initial map of the target scene by adding new elements to it at runtime [5,6,7]. The logical step forward of such methods is represented by markerless algorithms that are agnostic of any a-priori knowledge of the scene [8]. These methods are generally more complex and they often require more processing power than marker- or model-based solutions. Model-based methods, often regarded as a subset of markerless tracking, rely on the recursive or non-recursive detection of a 3D object in the scene with known shape and size [9].

On the other hand, well-established optical tracking methods based on fiducial markers are highly efficient, robust, and accurate [10] and yet they are very sensitive to occlusions and they are rather obtrusive, particularly for restricted work-spaces.

Irrespective of the method adopted, the ideal tracking method should be computationally efficient, it should be resilient to ambiguities resulting from markers occlusions or inconsistent lighting conditions, and it should provide accurate localization results.

Nowadays, AR headsets are the leading edge and the major output medium of AR technology for a broad range of potential applications due to the success of consumer-oriented models such as the Microsoft HoloLens (Microsoft Corporation, Redmond, WA, USA) [11,12]. AR headsets, commonly referred to as head-mounted displays (HMDs), represent the most ergonomic AR solution since they are capable to seamlessly blend real world and virtual elements whilst retaining the user’s natural and egocentric viewpoint.

This feature has been stimulating massive efforts and resources, both among researchers and companies, to push the technology towards the implementation of wearable devices capable to offer efficient self-tracking and advanced rendering capabilities without any auxiliary equipment.

Recently, the increasing capability of computing power and mobile graphics has made possible the release of self-contained wearable AR platforms featuring efficient head-anchored tracking systems (i.e., “inside-out”) that significantly increase the overall portability. The progress in terms of latency and tracking robustness was achieved by deploying novel strategies that combine data gathered from optical and inertial sensing devices. Such hybrid tracking modalities are intended to counter some of the drawbacks of purely optical self-tracking techniques that rely on wearable head-anchored stereo cameras.Here follows a list of the most relevant ones:

- the presence of poorly calibrated tracking cameras;
- the anthropomorphic geometry of the head-anchored stereo rig [13,14,15] (e.g., the short baseline of the stereo cameras, which should be ≈ to the user’s interpupillary distance, and the limited camera focal length that should comply with orthostereoscopic viewing conditions);
- the presence of inaccuracies in the feature detection that may lead to numerical instability and tracking ambiguities particularly for those tracking strategies that rely on a reduced number of feature points;
- the limited frame rate of the tracking cameras typically mounted over AR headsets (60 Hz at most);
- the presence of noise due to head movements affecting the quality of the tracking;
- the presence of occlusions on the line-of-sight between the user’s wearing the AR headset and the target scene;
- the latency typical of purely optical tracking methods that results in misregistration between virtual content and real world in optical see-through (OST) headsets or in delayed perceptions of the reality in video see-through (VST) HMDs [16].

On the other side, the tracking based on inertial measurement units (IMUs) is intrinsically insensitive to occlusions, variable lighting conditions, and it can run at fast sampling rates (up to 1000 Hz).

In previous works, we introduced the use of different custom-made VST headsets for surgical guidance [17,18,19]. In such applications, the locational realism of the AR scene is of paramount importance for whose achievement the virtual content of the scene must be observed by a couple of virtual viewpoints that mimic the real cameras in terms of intrinsic and extrinsic parameters. In those works, the estimation of the extrinsic parameters was based on inside-out tracking techniques that relied on the stereo localization of a set of three monochromatic markers followed by an iterative perspective-3-point-based pose computation [20].

Unfortunately, the above-cited drawbacks limit the efficacy of such head-anchored optical tracking approach in actual applications. To counter these limitations and to alleviate the ambiguity and the instability of the perspective-3-point (P3P) pose computation, in this paper we present a heterogeneous tracking method (optical + inertial) suited for AR headsets. The hybrid tracking method exploits Kalman Filter (KF) algorithm to combine the results of the optical tracking with the fast rotational response of an IMU. This results in a ubiquitous tracking platform which recovers easily from dynamic occlusions and tracking failures due to the ambiguities of the optical tracking with a minimum set of markers (i.e., three) [21].

The algorithm was tested on a poorly-calibrated custom-made see-through HMD operating under uncontrolled lighting conditions, specifically conceived for high-precision manual tasks in a surgical or industrial scenario, and capable of providing both video and optical see-through-based augmentations [2].

The remainder of this paper is structured as follows: Section 2 surveys some of the most relevant works in the field of heterogeneous tracking specifically for AR applications. A detailed description of the hardware and software components is provided in Section 3.1 and Section 3.2. Section 3.3 illustrates the technical implementation of the calibration procedure. Section 3.4 outlines the sensor fusion algorithm based on a Kalman filter scheme. Section 4 reports the methods for validating the tracking method and discusses the results. Finally, Section 5 concludes with a summary and future work directions.

## 2. Related Works

Research on sensor fusion technology covers a broad range of potential applications from computer vision to robotics and gait analysis. A significant amount of research has been carried out in the last years to specifically support AR applications through sensor fusion strategies so as to compensate the drawbacks of individual tracking methods.

State et al. were the first to propose a solution integrating optical and magnetic tracking for different experimental AR systems [22]. The authors showed that the magnetic tracker was particularly useful in accelerating the image processing for feature extraction, in countering the ambiguity of the optical tracking, in compensating for the registration loss due to landmarks occlusions, and finally in improving the overall stability of the optical tracking.

In 2000, Yokokohji et al. [23] proposed an interesting hybrid method for accurate AR image overlay on VST HMDs based on vision-based tracking and accelerometers. In their method based on a “loosely coupled” extended Kalman filter (EKF), linear and angular accelerations were used for predicting the head motion with the aim to compensate the end-to-end system latency, making the optical tracking more robust when the user moves his/her head quickly.

In 2001, Satoh et al. [24] presented a hybrid tracking approach for outdoor AR applications with a custom-made VST HMD in which a head-anchored gyroscope provided the orientation of the user’s head. In their solution, the vision-based natural feature detection algorithm was used for gyroscope drift compensation rather than for fully integrating optical and inertial tracking data.

A more integrated strategy was proposed in 2004 [25] in which gyroscope data and model-based optical tracking data were fused by a heuristic assessment process and an EKF for pose recovery in outdoor AR applications. The gyroscope measurements helped predicting the orientation of the image line position from frame to frame and to counter possible line occlusions, whereas the optical tracking helped compensating for the gyroscope drift.

In 2002, Klein et al. [26] presented an integrated tracking strategy for pose recovery through the fusion of visual and inertial sensors. The gyroscope was used not only to predict the camera pose but also to provide a real-time estimate of the motion blur corrupting camera image measurements so to tune dynamically the parameters used for the feature detection algorithm.

In 2008, Bleser et al. [27] proposed a solution integrating markerless model-based optical tracking and inertial tracking (gyroscope and accelerometer) measurements in an EKF scheme for orientation and position estimation in AR and Virtual reality applications. They validated the tracking efficacy of their solution under controlled and real-world environments (with varying lighting conditions) and compared methods using only gyroscopes for camera orientation estimate and with methods exploiting the accelerometer measurements for camera position estimate.

In their work, Ercan et al. [28], observed via simulations that the tight combination, through an EFK framework with a “tightly coupled approach”, of optical tracking data (retrieved from an head-anchored camera) and accelerometer measurements used as control inputs did not seem to suffer from performance loss compared to fusing both camera and accelerometer measurements in the updated stage of the EKF. This finding suggests that the use of lower complexity EKFs may not affect tracking accuracy.

A hybrid tracking solution comprising a single camera, an IMU with 3-axis gyroscope and accelerometer, and a Global Positioning System (GPS) unit was proposed in 2012 [29] for AR applications with a VST helmet. In indoor applications the authors demonstrated the accuracy and robustness of the tight combination of the IMU and optical tracking data through an EKF framework is comparable to more complex solutions based on stereo tracking platforms and GPS units.

In 2014, Menozzi et al. [30] presented the development of an integrated pose estimation solution suited for AR helmets in outdoor environments integrating input data from IMU, helmet-mounting tracking camera, and GPS in an EKF framework. In the work, the authors demonstrated the accuracy and robustness of the whole pose estimation process compared to off-the-shelf GPS-based inertial navigation systems, even in the presence of magnetic disturbance.

Likewise, He et al. [31] proposed an ego-motion hybrid tracking method based on visual-inertial sensors embedded in the Google glass for blind indoor navigation. As in the previous solutions, an EKF framework employed to fuse visual and inertial estimations improved the robustness of pose estimation in highly dynamic environments.

Finally in 2018, Qian et al. [32] proposed a wearable OST display equipped with a helmet-mounted stereo cameras and an inertial sensor for SLAM and navigation guidance specifically designed for outdoor inspection tasks. For this purpose, the authors proposed an elaborated detection process that compares, at runtime, the observed scene with an abstract metric-topological map of the outdoor workspace. The map was built through on deep convolutional neural networks that exploits the combination of stereo cameras and IMU measurements. This approach ensured the robustness of the scene-matching under different weather/lighting conditions.

## 3. Materials and Methods

This section provides a detailed description of the hardware and software components. The following notation is used throughout the paper. Uppercase typewriter letters denote spatial coordinate systems, such as the tracking camera coordinate system C. Lowercase letters denote scalar values such as the camera focal length *f* or the reprojection residual. Vectors are denoted by lowercase bold letters with a superscript denoting the reference coordinate system (e.g., a 3D point in camera coordinates $v^{C}$ or a 2D image point in camera image coordinates $p^{S}$). Vectors can also be expressed in component form, with a bold subscript indicating the correspondence (e.g., $v^{C}={\left(x_{v},y_{v},z_{v}\right)}^{C}$). Matrices are denoted by uppercase letters (e.g., the intrinsic camera matrix of camera ${}_{}^{C}K$). The 6 DoF transformations from one coordinate system to another are defined as follows: given two coordinate systems A and B, the transformation from A to B is ${}_{A}^{B}T={(}_{A}^{B}R{,}_{A}^{B}t)$ where ${}_{A}^{B}R$ is the rotation matrix and ${}_{A}^{B}t$ is the translation vector. Therefore, we have:

(1) $$v^{B}{=}_{A}^{B}Rv^{A}{+}_{A}^{B}t$$

The custom-made see-through HMD together with the AR software framework for the deployment of the AR application were recently presented in [2]. In the following two subsections, we briefly summarize the main features of the wearable AR platform.

### 3.1. Hardware

**Figure 1:**
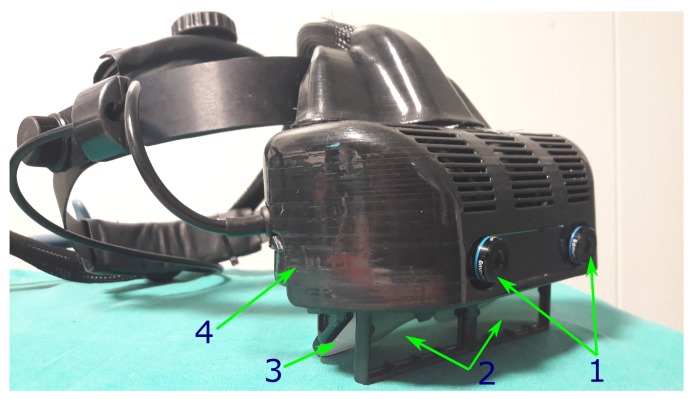
The custom-made hybrid video/optical see-through head-mounted display. 1→Pair of stereo cameras for the inside-out optical tracking and the camera-mediated view. 2→Pair of liquid-crystal (LC) optical shutters for the video-optical switching mechanism. 3→Beam combiners of the see-through display. 4→Plastic frame that holds all the components around the see-though visor.

The see-through AR headset was designed and assembled by re-engineering and reworking a commercial binocular OST visor (ARS.30 by Trivisio [33]) with a similar approach of our previous works [34,35] (Figure 1). The main characteristics of the headset are listed below.

The custom-made headset is capable to provide both optical and video see-through-based augmentations through a mechanism that relies on a pair of liquid-crystal (LC) optical shutters (FOS model by LC-Tec) stacked over the beam combiners of the OST visor. The transparency of the see-through display can be controlled by modifying the light transmittance of the two LC panels through an externally applied drive voltage. This enables switching between the unaided binocular view (i.e., OST modality with shutters off) and a camera-mediated view (i.e., VST modality with shutters on).

The ARS.30 visor is provided with dual SXGA OLED panels with 1280×1024 resolution, a diagonal field of view (FOV) of $30^{\circ }$ and an eye-relief of 3 cm each. The resulting angular resolution of the OST display is ≈1.11 arcmin/pixel. The collimation optics of the visor was re-engineered to offer a focal length of about 50 cm. The two optical engines of the visor are slightly toed-in: this means that the optical axes of the two displays are made to converge to approximately the focal length of the collimation optics. These last two features represent a defining and original feature to mitigate the vergence-accommodation conflict and the focus rivalry when the headset is used for close-up works (i.e., to aid high-precision manual tasks).

The visor is housed in a custom-made plastic frame whose function is to incorporate the two LC shutters and to act as support for the pair of front-facing RGB cameras. The stereo cameras pair is composed by two USB 3.0 LI-OV4689 cameras by Leopard Imaging, both equipped with 1/3’’ OmniVision CMOS 4M pixels sensor.

The camera pair is also toed-in and mounted on the top of the visor with an anthropometric interaxial distance (≈6.3cm) to mitigate the effect of the camera-to-eye parallax. By doing so, we aim to prevent substantial distortions in the patterns of horizontal and vertical disparities between the stereo cameras frames presented on the displays of the headset, and therefore we try to pursue a quasi-orthostereoscopic perception of the scene under VST view without any perspective conversion of the camera frames [36]. Both cameras are equipped with a M12 lens whose focal length (f = 6 mm) ensures sufficient stereo overlap of the camera frames in the peripersonal space.

Even though in this study we validated the heterogeneous tracking method with the HMD working in VST modality the proposed strategy is not limited to VST HMDs.

**Figure 2:**
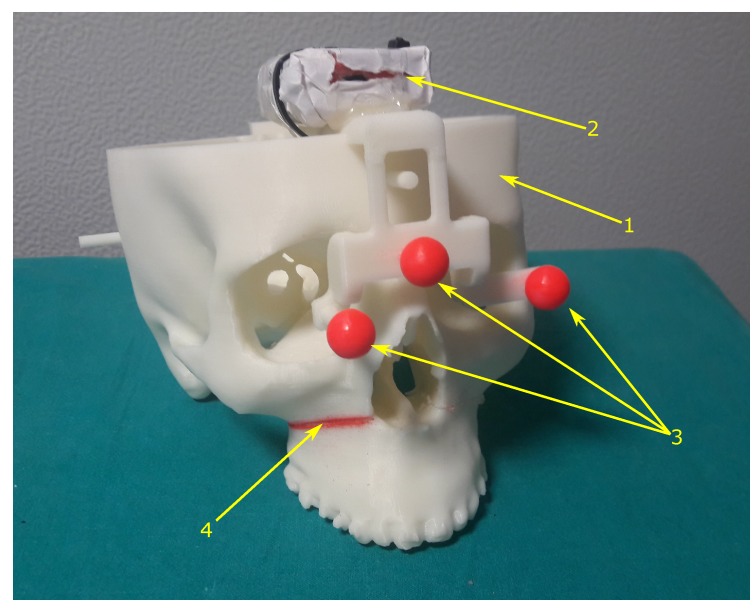
The 3D printed replica of the human skull used in the experimental session as target scene to validate the heterogeneous tracking performance. 1→The 3D printed replica of the human skull. 2→The inertial measurement unit (IMU) anchored to the skull replica 3→The spherical markers of the optical frame. 4→The red-dyed fracture (a Le Fort 1 osteotomy) considered as reference feature for the assessment of the virtual-to-real overlay accuracy.

The inertial measurment unit (IMU) is a wireless unit (MTw by XSens): it incorporates a 3D linear accelerometer, a 3D gyroscope, and a 3D magnetometer. The IMU performs real-time signal elaboration and transmits the sensor orientation (Euler angles, quaternions or rotation matrices) and raw sensor data (acceleration vector, angular velocities, magnetic field vector). In our experiments, we collected the IMU orientation and raw signals at a sampling rate of 120 Hz, whereas the frame rate of the stereo cameras was of 60 frames-per-second (fps). The IMU was anchored on the frame of optical markers used for the inside-out tracking (Figure 2).

### 3.2. AR Software Framework

The AR software framework for surgical guidance was thoroughly described in a recently published paper [2]. Here, we recall its main features:

- The software is capable of supporting the deployment of AR applications on different headsets (both VST and OST HMDs) and it features a non-distributed architecture, which makes it compatible with embedded computing units.
- The software framework is based on Compute Unified Device Architecture (CUDA) in order to harness the power of parallel computing over the GPU cores of the graphic card. This architecture makes the software framework computationally efficient in terms of frame rate and latency: the average frame rate of the AR application is ≈30 fps.
- The software is suited to deliver in situ visualization of medical imaging data, thanks to the employment of the open-source computer library VTK for 3D computer graphics, modelling, and volume rendering of medical images [37].
- The software framework is highly configurable in terms of rendering and tracking capabilities.
- The software can deliver both optical and video see-through-based augmentations.
- The software features a robust optical self-tracking mechanism (i.e., inside-out tracking) based on OpenCV API 3.3.1 [38], that relies on the stereo localization of a set of spherical markers (i.e., the optical frame), as described in more details in the next subsection.

#### Optical Inside-Out Tracking Algorithm

The optical self-tracking method relies on the stereo localization of a set of three identical markers attached to the target object.

Although tracking by detection can be more stable and accurate using a greater number of features [39], reducing the number of reference points to three represents a valuable feature with regard to computational efficiency of the tracking algorithm. In addition, reducing the number of tracking markers is particularly important for those applications that demand for a reduced logistic impact in the setup phase and that require for limited line-of-sight constraints (e.g., in surgical or industrial applications) [2].

The optical tracking method delivers the pose of the optical frame, whose coordinate system is F, with respect to the left tracking cameras (C). Hereafter, we use the term camera pose to refer to such pose. The algorithm can be broken down into four main stages:

1. Markers detection.
2. Stereo matching.
3. First stage of the camera pose estimation through the unambiguous closed-form solution of the absolute orientation problem with three points (i.e., estimation of the rigid transformation that aligns the two sets of corresponding triplets of 3D points). Hereafter, we label this pose as ${\mathrm{OT}}_{\mathrm{noref}}$.
4. Second stage of the camera pose estimation through an iterative optimization method. Hereafter, we label this pose as ${\mathrm{OT}}_{\mathrm{ref}}$.

In the first stage, the markers’ centroids are detected onto the image planes of the stereo cameras through Hue-Saturation-Value (HSV) color space segmentation and blob detection. Next, the stereo correspondence is solved by applying epipolar geometry rules. Then, after stereo triangulation, ${\mathrm{OT}}_{\mathrm{noref}}$ is computed through a least-squares fitting method [40]. Notably, the stereo setting makes the absolute orientation problem unambiguously solvable in a closed-form fashion also with three reference markers. Nevertheless, and as explained in more details in [41] and in [42], the anthropomorphic geometry of our stereo setting (i.e., the short baseline *b* and the limited focal length *f* of the tracking cameras) can ensure accurate stereo tracking of the markers only at close distances. By way of illustration, given a disparity error of Δd, the main component of the error is measured along the depth-axis (Δz) and is calculated as follows:

(2) $$\Delta z=\frac{z^{2}}{\mathrm{fb}}\Delta d$$

This localization error is particularly sensitive to the disparity accuracy yielded by the feature extraction algorithm and to the calibration errors in estimating the intrinsic and extrinsic camera parameters of the stereo pair of cameras.

In particular, a relevant drawback of using wearable trackers is represented by the non-ideal stability in the constraints between the two stereo cameras, which may cause a potential change in their relative pose over time [13]. Such systems need frequent calibration to cope with degradation of the stereo calibration over time, particularly when more complex non-automatic self-calibration methods are adopted [43].

For this reason, an iterative optimization step, which refines the pose of each camera separately, is required to achieve sub-pixel AR registration accuracy on both camera image planes. This final task, commonly referred to as Perspective-*n*-Point (P*n*P) problem in computer vision [44] and exterior orientation or space resection problem in photogrammetry, computes ${\mathrm{OT}}_{\mathrm{ref}}$ given the intrinsic parameters of the camera and a set of *n* world-to-image point correspondences. Notably, three is the minimum number of markers that ensures a finite number of solutions for the P*n*P problem [45,46]. A useful overview of the state-of-the-art methods for solving the P*n*P problems can be found in [47] and in [48].

The optimization algorithm solves iteratively the PnP problem for both cameras by minimizing a cost function formulated as the sum of the square measurement error (reprojection residuals $d_{i}$) between detected image points $p_{i}^{S}$ and calculated projections $\hat{p_{i}^{S}}$ of the corresponding world points ($P_{i}^{W}$):

(3) $${\left[{\;}_{F}^{C}{R|}_{F}^{C}t\;\right]}_{\mathrm{refined}}=\underset{j}{arg min}\sum_{i=1}^{3}d{\left(p_{i}^{S},\;\hat{p_{i}^{S}}\right)}^{2}=\underset{j}{arg min}\sum_{i=1}^{3}{∥p_{i}^{S},\;\hat{p_{i}^{S}}({}_{}^{C}K,\hat{R},\hat{t},P_{i}^{F}∥}^{2}$$

Where ${}_{}^{C}K$ is the matrix of intrinsic parameters of the calibrated camera and $\hat{R}$, $\hat{t}$ are the rotation matrix and translation vector to be optimized.

The method is based on a non-linear iterative Levenberg–Marquardt optimization. Unfortunately, this technique relies on a good initial guess to converge to the correct solution and, in case of noisy data or unreliable calibration measurements, there is no guarantee that the algorithm will eventually converge or that it will converge to the correct solution [49]. Therefore, the problem of iteratively optimizing the camera pose from a set of 3D to 2D point correspondences suffers from numerical instability and, in case of three reference markers, it can deliver up to four ambiguous solutions. In addition, some multiple solutions are close to each other, and this occurrence may be a cause of gross errors by selecting incorrect solutions. The multi-solution phenomenon typical of the P3P problem is particularly significant for those real-world settings in which the scene object is under non-controllable and noisy lighting conditions. In this case, the feature extraction may yield unreliable results in terms of image coordinates of the markers’ centroids, hence it can result in inaccurate and unstable tracking data even if we added more reference markers.

In light of this, with this work we aim to improve the reliability and robustness of the self-tracking algorithm by querying also the IMU data when computing motion correlation. The fusion of optical and inertial tracking data can help improving the tracking accuracy and robustness by capturing the orientation of the scene object also when some of the markers are occluded and/or when the optical tracking yields unstable and ambiguous results.

In the next subsection, we will describe the calibration procedure needed for the orientation alignment between the local IMU reference system (I) and the optical frame reference system (F).

### 3.3. Calibration Procedure for Orientation Alignment of Inertial and Optical Coordinate Systems

**Figure 3:**
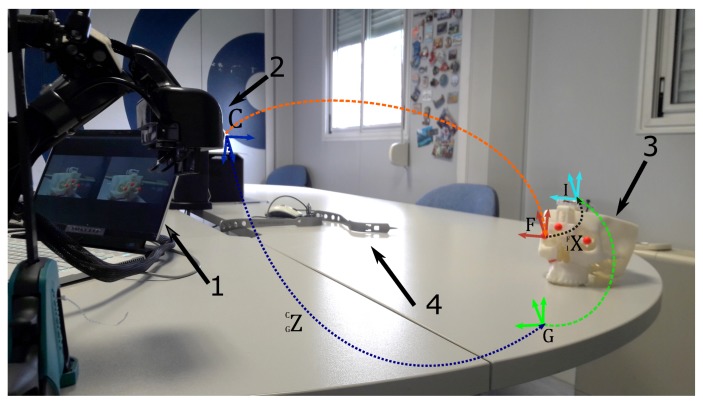
Experimental setup for the calibration procedure and the experimental session. 1→ External laptop running the augmented reality (AR) application with the side-by-side augmented camera frames. 2→ The custom-made see-through head-mounted display (HMD) capable of providing both video and optical see-through-based augmentations. 3→ The 3D-printed replica of a human skull comprising the IMU and the optical frame. 4→ Non-controllable and noisy lighting conditions.

**Figure 4:**
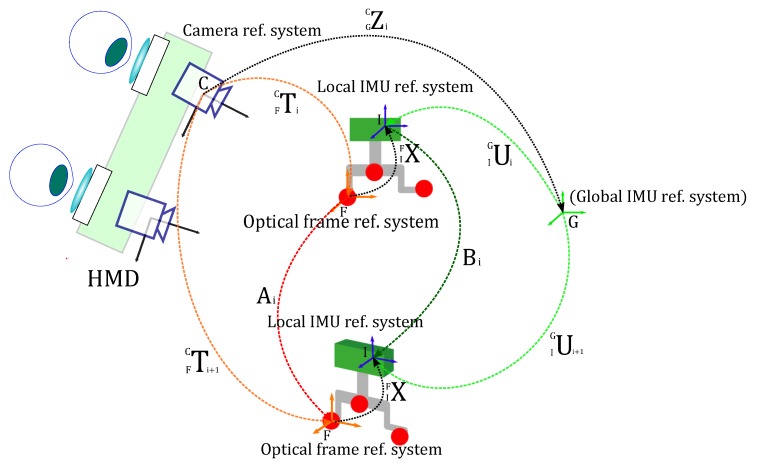
Schematics of the transformations involved in the AX = XB calibration procedure to estimate the orientation between optical frame and IMU.

The Figure 3 shows the experimental setup. All the reference systems and the transformations involved in the calibration procedure are depicted in Figure 4. Both the optical frame and the IMU are anchored to a 3D-printed replica of a human skull used for simulating an intervention of maxillofacial surgery. The skull replica presents an artificial fracture (i.e., a Le Fort 1 osteotomy) on the right side, and it embeds the three spherical markers that define the optical frame coordinate system. To counter the limitation of using visible light as source of information for the optical tracking, the markers were dyed in fluorescent red, since fluorescent dyes peak the S channel of the HSV color space and boost the response of the camera CMOS sensor [2,50]. In our setup the IMU is attached to the optical frame. For this reason, the headset with the embedded stereo cameras pair is assumed to remain stationary during both the calibration procedure and the validation tests. Note that the proposed method would also apply to setups where the IMU is anchored to the headset [51]. In that case, it would be the scene object (i.e., the skull replica) to be assumed stationary during the calibration.

The intrinsic (linear and non-linear) and extrinsic parameters of the stereo cameras were estimated through a standard camera calibration routine [4]. This procedure was performed using the MATLAB camera calibration toolbox (R2018b MathWorks, Inc., Natick, MA, USA), immediately prior to the procedure for estimating the orientation alignment between optical frame and IMU. Notably, the validation tests were then performed without conducting a prior-to-use ad hoc calibration of the stereo cameras, after several days of extensive use of the headset. By doing so, we intended to simulate a common use case outside laboratory environment.

For the problem of computing the rotation matrix that aligns I to F (i.e., hand–eye calibration or “AX=XB” problem), we adopted the closed-form least squares solution proposed by Park et al. [52]. The calibration procedure requires performing a series of n arbitrary movements of the 3D-printed replica of the human skull with respect to the stationary HMD. In our experiments, we considered a number of arbitrary calibration poses of 10 to 14 (n=10-14). For each i pose, the optical tracking data encapsulating the orientation of F with respect to C ($T_{i}$) and the inertial tracking data encapsulating the orientation of I with respect to G ($U_{i}$), were recorded and stored.

The hand–eye calibration problem is represented by the homogeneous matrix equation of the well-known form:

(4) $$A{(}_{I}^{F}X)={(}_{I}^{F}X)\;B$$

Where:

- Given n-1 pairs of consecutive arbitrary poses between the optical frame reference system F and the tracking camera reference system C, A is the rotation matrix that describes the relative orientation between each pair.
- Given n-1 pairs of consecutive arbitrary poses between the local IMU reference system I and the global IMU reference system G, B is the rotation matrix that describes the relative orientation between each pair.
- ${}_{I}^{F}X$ is the unknown rotation matrix between the I and F.

During the calibration procedure, for each two consecutive arbitrary poses of the scene object, the tracking data are recorded for 5 s in static conditions. The tracking data are expressed in terms of quaternions are then averaged [53]: we considered for each static pose the maximum likelihood estimate of the average quaternion as the eigenvector associated to the maximum eigenvalue of the matrix formed by the weighted quaternions [54].

In Figure 4, $A_{i}$ denotes the motion between the poses $T_{i}$ and $T_{i}+1$. Similarly, $B_{i}$ denotes the motion between $U_{i}$ and $U_{i}+1$.

Equation (4) can then be rewritten as:

(5) $$A_{i}{(}_{I}^{F}X)={(}_{I}^{F}X)B_{i};\;\;i=1...n$$

$A_{i}$ and $B_{i}$ can be computed as:

(6) $$\begin{array}{c} A_{i}={{(}_{F}^{C}T_{i}^{-1})}_{F}^{C}T_{i+1}\\ B_{i}={{(}_{I}^{G}U_{i}^{-1})}_{I}^{G}U_{i+1} \end{array}$$

Where:

- ${}_{F}^{C}T_{i}$ and ${}_{F}^{C}T_{i+1}$ are the orientation of the optical frame F with respect to the tracking camera C in terms of rotation matrices, with the scene object at the i and i+1 pose respectively. These tracking data are recorded by querying the tracking camera.
- ${}_{I}^{G}U_{i}$ and ${}_{I}^{G}U_{i+1}$ are the orientation of the IMU I with respect to the global inertial reference system G in terms of rotation matrices, with the scene object at the i and i+1 pose respectively. These tracking data are recorded by querying the IMU sensor.

Once ${}_{I}^{F}X$ is calculated, the rotation matrix ${}_{G}^{C}Z_{i}$ between the tracking camera C and the global IMU reference system G is easily derived for every pose i as follows:

(7) $$\begin{array}{c} {}_{G}^{C}Z_{i}={{(}_{F}^{C}T_{i})}_{I}^{F}X{(}_{I}^{G}U_{i}^{-1}) \end{array}$$

To minimize the error, ${}_{G}^{C}\bar{Z}$ is computed by averaging ${}_{G}^{C}Z_{i}$ values over the i poses. As a result, the orientation of the scene object with respect to the camera can be indirectly estimated at any instant by querying the IMU data with the following equation:

(8) $$\begin{array}{c} {}_{F}^{C}R_{\mathrm{IMU}}\left(\theta_{x},\theta_{y},\theta_{z}\right)={{(}_{G}^{C}\bar{Z})}_{I}^{G}U{(}_{I}^{F}X^{-1}) \end{array}$$

### 3.4. Sensor Fusion Based on Kalman Filter

**Figure 5:**
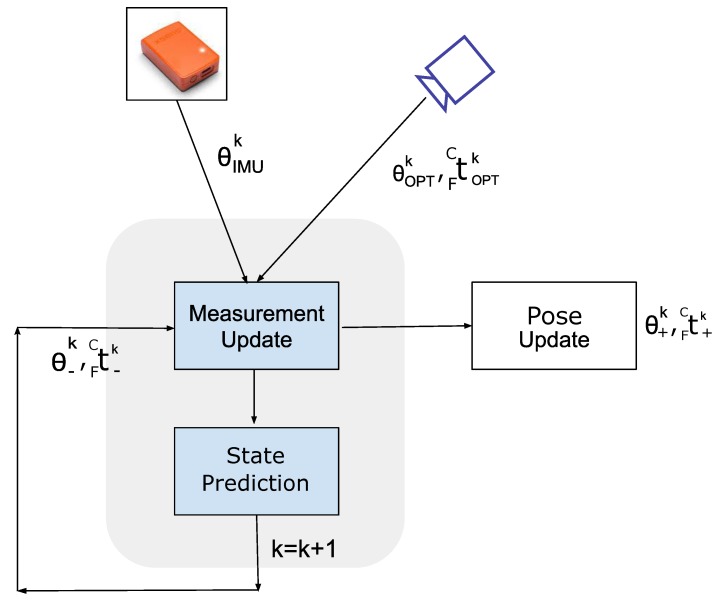
Block Diagram of the Kalman Filter algorithm.

A standard linear Kalman filter (KF) was implemented to estimate the correct pose of the target object and mitigate the impact of dynamic occlusions and tracking failures by fusing the information of the inertial tracking data and the optical tracking data (Figure 5).

In order to integrate IMU time series with those of the optical tracking, the former were down-sampled by a factor of two and synchronized with the optical tracking series through cross-correlation.

The pose to be estimated is represented by two components: the Euler rotation angles ($\theta_{x}^{k}$, $\theta_{y}^{k}$, $\theta_{z}^{k}$) associated to ${}_{F}^{C}R^{k}$ and the translation vector ${}_{F}^{C}t^{k}={\left(x_{t},y_{t},z_{t}\right)}^{C}$, both considered at any k instant. For the implementation of the Kalman filter we defined the following relations: the state $x_{k}$ is a 12x1 vector state defined as:

(9) $$x_{k}={\left[x_{t},y_{t},z_{t},{\dot{x}}_{t},{\dot{y}}_{t},{\dot{z}}_{t},\theta_{x},\theta_{y},\theta_{z},{\dot{\theta }}_{x},{\dot{\theta }}_{y},{\dot{\theta }}_{z}\right]}_{k}$$

The measurement vector is a 9x1 vector represented by the translation along x,y,z of the optical tracking signal and by the rotation evaluated by the inertial and optical system. The $y_{k}$ at instant k is defined as:

(10) $$y_{k}=\left[{\left(x_{t}\right)}_{\mathrm{OPT}},{\left(y_{t}\right)}_{\mathrm{OPT}},{\left(z_{t}\right)}_{\mathrm{OPT}},{\left(\theta_{x}\right)}_{\mathrm{IMU}},{\left(\theta_{y}\right)}_{\mathrm{IMU}},{\left(\theta_{z}\right)}_{\mathrm{IMU}},{\left(\theta_{x}\right)}_{\mathrm{OPT}},{\left(\theta_{y}\right)}_{\mathrm{OPT}},{\left(\theta_{z}\right)}_{\mathrm{OPT}}\right]$$

The discrete-time state transition imposed by the Kalman filter is expressed through the following two relations (i.e., the process model and the measurement model):

(11) $$\begin{array}{c} x_{k}=Ax_{k}-1+q_{k}\\ y_{k}=Hx_{k}+v_{k} \end{array}$$

In the process model equation, the state transition matrix A relates the state at the previous time frame k-1 to the current frame at instant k.

(12) $$A=\left[\begin{array}{cccccccccccc} 1 & 0 & 0 & dT & 0 & 0 & 0 & 0 & 0 & 0 & 0 & 0\\ 0 & 1 & 0 & 0 & dT & 0 & 0 & 0 & 0 & 0 & 0 & 0\\ 0 & 0 & 1 & 0 & 0 & dT & 0 & 0 & 0 & 0 & 0 & 0\\ 0 & 0 & 0 & 1 & 0 & 0 & 0 & 0 & 0 & 0 & 0 & 0\\ 0 & 0 & 0 & 0 & 1 & 0 & 0 & 0 & 0 & 0 & 0 & 0\\ 0 & 0 & 0 & 0 & 0 & 1 & 0 & 0 & 0 & 0 & 0 & 0\\ 0 & 0 & 0 & 0 & 0 & 0 & 1 & 0 & 0 & dT & 0 & 0\\ 0 & 0 & 0 & 0 & 0 & 0 & 0 & 1 & 0 & 0 & dT & 0\\ 0 & 0 & 0 & 0 & 0 & 0 & 0 & 0 & 1 & 0 & 0 & dT\\ 0 & 0 & 0 & 0 & 0 & 0 & 0 & 0 & 0 & 1 & 0 & 0\\ 0 & 0 & 0 & 0 & 0 & 0 & 0 & 0 & 0 & 0 & 1 & 0\\ 0 & 0 & 0 & 0 & 0 & 0 & 0 & 0 & 0 & 0 & 0 & 1 \end{array}\right]$$

where dT is the discrete time interval between two consecutive frames (i.e., dT=1/60Hz=0.0167 s). In the measurement model equation, the measurement matrix H maps the state vector $x_{k}$ to the measurement vector $y_{k}$, whereas $q_{k}$ and $v_{k}$ are random variables (white noise) associated to the process and measurement noise covariance matrices Q and V.

(13) $$H=\left[\begin{array}{cccccccccccc} 1 & 0 & 0 & 0 & 0 & 0 & 0 & 0 & 0 & 0 & 0 & 0\\ 0 & 1 & 0 & 0 & 0 & 0 & 0 & 0 & 0 & 0 & 0 & 0\\ 0 & 0 & 1 & 0 & 0 & 0 & 0 & 0 & 0 & 0 & 0 & 0\\ 0 & 0 & 0 & 0 & 0 & 0 & 1 & 0 & 0 & 0 & 0 & 0\\ 0 & 0 & 0 & 0 & 0 & 0 & 0 & 1 & 0 & 0 & 0 & 0\\ 0 & 0 & 0 & 0 & 0 & 0 & 0 & 0 & 1 & 0 & 0 & 0\\ 0 & 0 & 0 & 0 & 0 & 0 & 1 & 0 & 0 & 0 & 0 & 0\\ 0 & 0 & 0 & 0 & 0 & 0 & 0 & 1 & 0 & 0 & 0 & 0\\ 0 & 0 & 0 & 0 & 0 & 0 & 0 & 0 & 1 & 0 & 0 & 0 \end{array}\right]$$

The Q matrix is

(14) $$Q=\left[\begin{array}{cccc} Q_{t} & 0 & 0 & 0\\ 0 & Q_{\dot{t}} & 0 & 0\\ 0 & 0 & Q_{\theta } & 0\\ 0 & 0 & 0 & Q_{\dot{\theta }} \end{array}\right]$$

where $Q_{t}$, $Q_{\dot{t}}$, $Q_{\theta }$ and $Q_{\dot{\theta }}$ are 3 by 3 diagonal matrices with equal diagonal elements ($q_{t}$, $q_{\dot{t}}$, $q_{\theta }$, $q_{\dot{\theta }}$, respectively). We fixed the process covariance to obtain a compromise between estimation accuracy and filter delay. The V matrix is in the form

(15) $$V=\left[\begin{array}{ccc} Q_{x_{OPT}} & 0 & 0\\ 0 & Q_{\theta_{IMU}} & 0\\ 0 & 0 & Q_{\theta_{OPT}} \end{array}\right]$$

where $Q_{x_{OPT}}$, $Q_{\theta_{IMU}}$ and $Q_{\theta_{OPT}}$ are 3 by 3 diagonal matrices with equal diagonal elements ($q_{x_{OPT}}$, $q_{\theta_{IMU}}$ and $q_{\theta_{OPT}}$) that we have estimated from 20 s of signals acquired in static conditions. Notably, we assumed the sensors noise associated to the optical translation, to the optical rotation, and to the IMU rotation, to be white and independent.

## 4. Experiments and Results

To validate the impact of the proposed KF-based integration of optical and inertial data in increasing the tracking accuracy under dynamic conditions, we conducted an experimental session with the HMD described in Section 3.1 and taking advantage of the AR software framework whose main features were listed in Section 3.2. The dynamic test was conducted under partial occlusion conditions, under rather inconsistent lighting conditions, and with poorly calibrated stereo cameras.

During the test, the 3D-printed replica of the human skull with the optical frame and the IMU anchored to it was moved around within the line-of-sight of the stereo cameras (Figure 10). The video stream of the stereo cameras was recorded at 60 fps for about 50 s. Therefore, the overall number of recorded stereo frames was of ≈3000. The orientation data of the IMU were recorded asynchronously at 120 Hz. By running the AR application with the recorded video stream of the stereo cameras, we were able to collect the pose of the target scene (i.e., the camera pose) determined through the optical tracking algorithm described in Section 3.2 for each recorded frame. All these poses (${}_{F}^{C}T_{\mathrm{OPT}}$) were so stored into a .txt file. The AR application was then suitably modified to directly query this .txt file instead of performing a real-time tracking.

In order to integrate IMU time series with those of the optical tracking the following three operations were undertaken:

1. The IMU data were down-sampled by a factor of two to match the sampling rate of the optical data.
2. Using the IMU data and the calibration data (see Section 3.3), the orientation of the target scene with respect to the tracking camera in terms of rotation matrices ${}_{F}^{C}R_{\mathrm{IMU}}$ was determined.
3. The two time series of the Euler angles associated to ${}_{F}^{C}R_{\mathrm{OPT}}$ and ${}_{F}^{C}R_{\mathrm{IMU}}$ were synchronized through cross-correlation.

**Figure 6:**
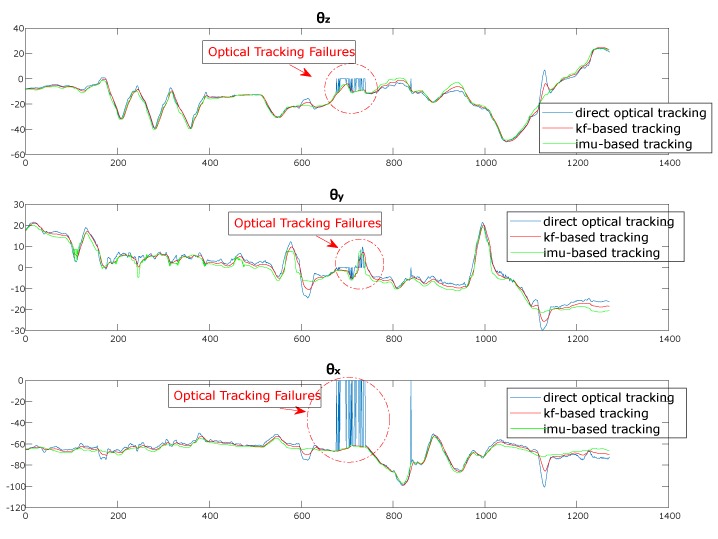
Time series of the Euler angles obtained the optical tracking, the inertial tracking, and the Kalman Filter-based heterogeneous tracking. For a better result display, only the first 1250 frames of the dynamic test are shown. Optical tracking failures are red circled. The trend of the Euler angles are at times rather different owing to the optical tracking ambiguities caused by variable lighting conditions and degraded tracking camera calibrations.

The two time series ${}_{F}^{C}T_{\mathrm{OPT}}$ and ${}_{F}^{C}T_{\mathrm{IMU}}$ were plugged into the KF framework to compute ${}_{F}^{C}T_{\mathrm{KF}}$. Both ${}_{F}^{C}T_{\mathrm{IMU}}$ and ${}_{F}^{C}T_{\mathrm{KF}}$ were stored in two .txt files as well. Figure 6 shows the Euler angles associated to ${}_{F}^{C}R_{\mathrm{OPT}}$, ${}_{F}^{C}R_{\mathrm{IMU}}$, and ${}_{F}^{C}R_{\mathrm{KF}}$.

We validated the efficacy of the proposed KF-based heterogeneous tracking by measuring the virtual-to-real overlay accuracy on the augmented frames. The augmented scene consisted of a virtual Le Fort 1 osteotomy line projected over the real osteotomy onto the maxillary bone of the skull replica. In order to facilitate the detection of the real osteotomy, the line was colored using red fluorescent dye (see next section).

A Video showing the comparison of AR overlay accuracy between non-refined optical tracking and refined KF-based optical-inertial tracking is provided as video file as Supplementary Materials.

### 4.1. Quantitative Evaluation of Virtual-to-Real Overlay Accuracy

In our quantitative evaluation, we compared four different tracking modalities: optical tracking without non-linear refinement (${\mathrm{OT}}_{\mathrm{noref}}$), optical tracking with non-linear refinement (${\mathrm{OT}}_{\mathrm{ref}}$), KF-based heterogeneous tracking with non-refined optical tracking data (${\mathrm{HT}}_{\mathrm{noref}}$), and KF-based heterogeneous tracking with refined optical tracking data (${\mathrm{HT}}_{\mathrm{ref}}$).

**Figure 7:**
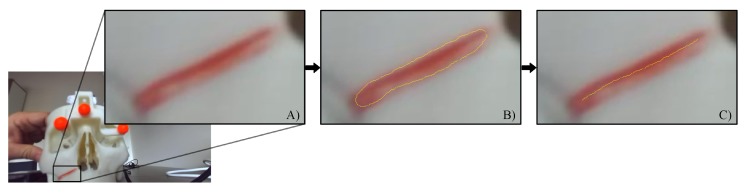
Feature detection on the real images: (**A**) Zoomed detail of the original real camera frame with the osteotomy line highlighted in red; (**B**) Result of the contour-based segmentation algorithm on the Hue-Saturation-Value colour space; (**C**) Final osteotomy center line detection.

**Figure 8:**
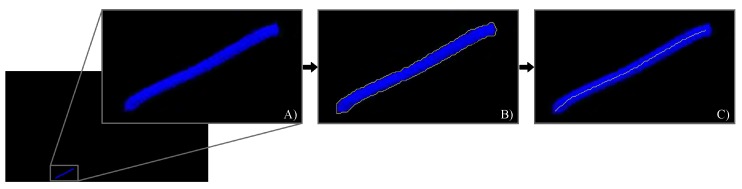
Feature detection on the virtual images: (**A**) Zoomed detail of the virtual osteotomy line; (**B**) Result of the threshold segmentation for boundary detection; (**C**) Final center line detection.

As anticipated, the goal of the quantitative evaluation was to measure, on the augmented frames, the overlay accuracy between real and virtual features (i.e., the real and virtual osteotomy lines). To this aim, we considered the Hausdorff distance between the two lines as a metric for measuring the “closeness” or the overlay error between them $o^{S}$ (Appendix A). The results and the statistical analysis was processed in MATLAB. The quantitative evaluation was broken down into the following steps:

- The images containing the left camera views of the real scene with the real osteotomy line were exported as a series of .png files with image resolution = camera resolution (1280 × 720). (Figure 7A).
- The associated virtual images with the virtual osteotomy line rendered as dictated by the tracking data for each modality, were exported as a series of .png files with the same image resolution as those associated to the real images (Figure 8A).
- The real images were converted in the HSV (hue, saturation, and value) color model to improve the robustness of the feature detection algorithm. The red fluorescent pigmentation used to highlight the real osteotomy peaks the S channel of the HSV color space and allowed the proper detection of structures that undergo non-uniform levels of illumination intensity, shadows and shading [50,55]. An active contour image segmentation technique was iteratively applied to the HSV-converted real image. Specifically, a coarse boundary was manually selected for the first image of the real group, and the detection algorithm autonomously adapted the contour to best fit the profile of the real osteotomy on the maxillary bone of the skull. The images were processed with no need of user’s input and using, as coarse outline, the one resulting from the previous iteration. The active contour algorithm follows the procedure described in [56]. This technique uses Mumford–Shah segmentation to stop the evolving curve on the desired boundary, offering positive results also in presence of smooth boundaries. The output of this first part of the processing is shown in Figure 7B. The resulting contour was then thinned into a line by removing pixels according to the algorithm described in [57]. This last step provides the center line illustrated in Figure 7C.
- The osteotomy line detection in the virtual images required a simpler approach than the previous one. As shown in Figure 8, the set of virtual images is characterized by a homogeneous black background. This prevented both the conversion to the HSV color model and the contour-based segmentation procedure. The latter is based on the assumption that the osteotomy line in the image does not undergo rapid or sudden displacements. This condition is satisfied for the set of real images sampled at 60 Hz, but it is not satisfied for those virtual images that may undergo “jerky” movements due to tracking inaccuracies/ambiguities, themselves caused by variable lighting conditions and degraded tracking camera calibrations. Each virtual image was therefore segmented using a standard threshold technique to obtain the boundary (as shown in Figure 8b). As in the case of the real images, the boundary is then thinned to obtain the center line highlighted in Figure 8c).
- The overlay error $o^{S}$ between real and virtual content was computed, for each pair of real and virtual frames, as the Hausdorff distance between the two center lines.

**Figure 9:**
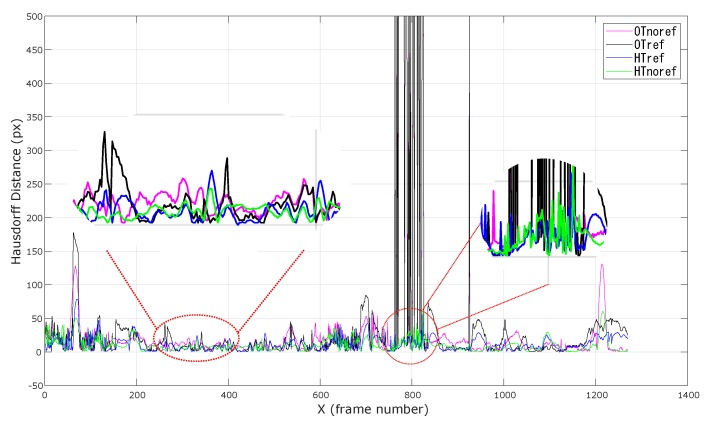
Overlay error measured as the Hausdorff distance between real and virtual detected features. For a better result display, only the first 1250 frames of the dynamic test are shown. The first zoomed circle on the left depicts how the Kalman filter (KF)-based heterogeneous tracking reduces the overlay error by improving the accuracy and robustness to ambiguities and tracking uncertainties. The second zoomed circle shows how the KF-based heterogeneous tracking is capable to tackle short-to-middle term optical tracking failures.

We performed two different analyses. In the first, the Hausdorff distances were measured for the four tracking modalities only for those augmented frames in which the optical tracking did not experience any tracking failure. In this way, we were able to isolate the improvement in tracking accuracy and robustness to ambiguities and tracking uncertainties directly associated to the KF-based integration of the inertial data (Figure 9).

In the second analysis, the same statistical indexes were computed also for those augmented frames in which the optical tracking experienced short-term tracking failures. We assumed short-term tracking failures those in which the optical tracking was not able to provide any orientation data for up to 0.5 s (i.e., 30 consecutive frames with no data from the optical tracking). With this analysis, we aimed to demonstrate the efficacy of the KF-based heterogeneous tracking in compensating short-to-middle term optical tracking failures.

### 4.2. Results and Discussion

As reported in Table 1 and Table 2, quantitative results are reported in terms of average value, standard deviation (Std Dev), median, and median absolute deviation (MAD) of the Hausdorff distances measured for all the tracking conditions.

**Figure 10:**
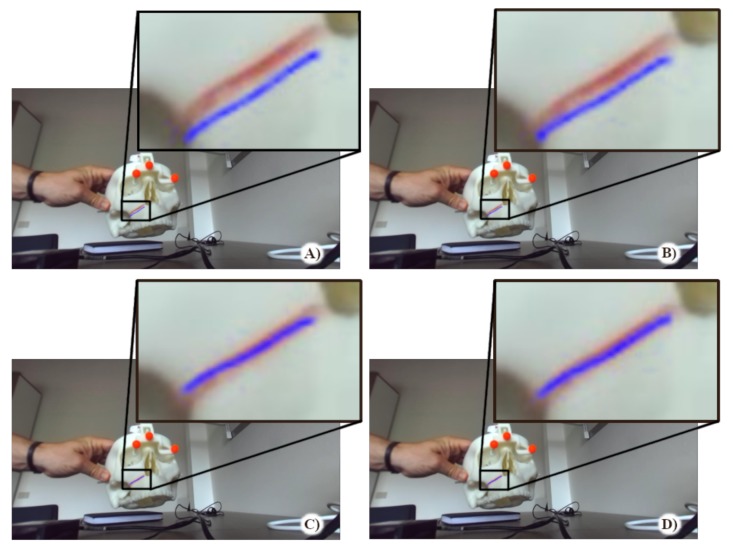
Augmented frames associated to the four tracking modalities:(**A**) Optical tracking without refinement; (**B**) Optical tracking with refinement; (**C**) Heterogeneous tracking without refinement; (**D**) Heterogeneous tracking with refinement.

The rather high values of the overlay error in both analysis is due to the particular metric used: the Hausdorff distance between two curves of different nature. This metric is intended to provide a “score” to the similarity between the two curves over the four different tracking modalities, rather than an absolute measure of alignment between them. To illustrate this, the AR overlay accuracy for all the tracking modalities appears to be generally higher than the figures provided in the two tables may suggest (Figure 10).

The quantitative results of the first analysis suggest that the KF-based tracking scheme improves the accuracy of the AR overlay by mitigating the effect of the tracking uncertainties and ambiguities due to inconsistent lighting conditions and/or poorly calibrated tracking cameras. Notably, in both the non-refined and refined tracking modalities the relative improvement in AR overlay accuracy is 33%. Table 1 shows that the non-linear camera pose refinement approach is rather sensitive to unreliable camera calibrations and/or inaccurate image data: the indexes of ${\mathrm{OT}}_{\mathrm{ref}}$ and ${\mathrm{HT}}_{\mathrm{ref}}$ are all greater than ${\mathrm{OT}}_{\mathrm{noref}}$ and ${\mathrm{HT}}_{\mathrm{noref}}$ due to the pose ambiguity resulting from the iterative solution of the P3P problem. This implies that in these conditions, the minimization of the reprojection residuals may fall into local minimum.

**Figure 11:**
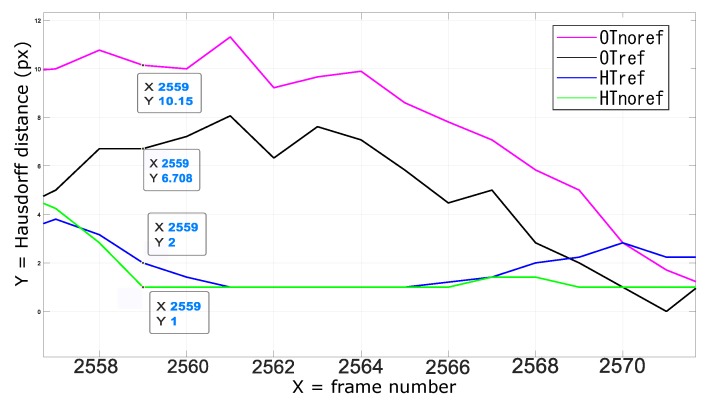
Overlay accuracy when ambiguity in perspective-3-point (P3P)-based optical tracking is solved.

Unsurprisingly, for those frames in which the pose refinement step finds out the global minimum, the overlay accuracy of ${\mathrm{OT}}_{\mathrm{ref}}$ and ${\mathrm{HT}}_{\mathrm{ref}}$ is higher than that obtained with ${\mathrm{OT}}_{\mathrm{noref}}$ and ${\mathrm{HT}}_{\mathrm{noref}}$ respectively (Figure 10 and Figure 11). Also here, the KF-based integration of the inertial data helps to increasing the tracking accuracy.

Finally, the results on the second analysis shown in Table 2 and in Figure 9 suggest that the KF-based tracking scheme is also capable to tackle short-to-middle term optical tracking failures due to partial occlusions.

As anticipated, the sensor noise associated to the optical and inertial data is assumed to be white and independent. In particular, the Euler angles associated to the inertial tracking, extracted by the proprietary XSENS algorithm, were considered as almost bias-free. As regards the optical tracking data, the white noise hypothesis may not be fully verified, particularly when the lighting conditions are not uniform. However, in most state-of-the-art PnP methods as the one we have used in our work, the data are assumed as noise-free [58].

## 5. Conclusions and Future Work

In this paper we present an heterogeneous KF-based tracking algorithm that improves the reliability and robustness of an optical tracking method that relies on a pair of stereo cameras and on the detection of a minimum set of reference markers. The KF framework integrates the orientation data of the optical tracking with the IMU data when computing motion correlation. Orientation alignment between optical frame and IMU reference frame is obtained by solving a standard hand–eye calibration method.

Our approach is conceived to be integrated in a custom-made AR headset that features ego motion estimation based on visible light stereo cameras.

This sensor fusion approach provides satisfactory results over short-to-middle term optical tracking failures and ambiguities. The high-frequency measurements provided by the IMU, together with the prediction stage typical of the KF framework, will substantially contribute in reducing the motion-to-photon latency and it will allow us to use the custom-made AR headset also under OST modality. Different PnP camera pose methods that assume no zero-mean Gaussian distribution in the feature tracking errors will be investigated, as well as possible alternatives such as particle filter-based tracking solutions [59,60,61].

Future work will also focus on performing an in-vitro study in AR-guided orthognathic surgery with the custom-made AR headset. We are therefore recruiting maxillofacial surgeons with different level of expertise in orthognathic surgery to test, on patient-specific replicas of the skull, AR-guided osteotomies of the maxillary bone. The goal of the study will be to compare the efficacy of the wearable AR platform in a real surgical scenario adopting two different tracking approaches: purely optical inside-out tracking and optical/inertial tracking.

Finally, another interesting line of research will focus on the integration of IMU-estimated position measurements in the KF framework to further improve the robustness and the efficacy of the self-tracking mechanism.

## Figures and Tables

**Table 1:** Results of the overlay accuracy analysis in pixels ($o^{S}$) without tracking failures.

Tracking Modality	Overlay Error (px)			
	Mean	Std Dev	Median	MAD
${\mathrm{OT}}_{\mathrm{ref}}$	17.83	21.1	11	14.1
${\mathrm{OT}}_{\mathrm{noref}}$	14.33	18.22	10	10.14
${\mathrm{HT}}_{\mathrm{ref}}$	11.96	11.28	8.6	8.29
${\mathrm{HT}}_{\mathrm{noref}}$	9.67	9.92	7	6.66

**Table 2:** Results of the overlay accuracy analysis in pixels ($o^{S}$) comprising sequence of camera frames with tracking failures (<30 consecutive frames).

Tracking Modality	Overlay Error (px)			
	Mean	Std Dev	Median	MAD
${\mathrm{OT}}_{\mathrm{ref}}$	69.46	150.45	13	93.9
${\mathrm{OT}}_{\mathrm{noref}}$	66.34	151.2	11.2	94.75
${\mathrm{HT}}_{\mathrm{ref}}$	19.58	30.57	9.96	17.44
${\mathrm{HT}}_{\mathrm{noref}}$	16.7	27.9	7.62	15.87

## Supplementary Materials

The following is available online at https://drive.google.com/file/d/1GCaV8guexBDEeX5NfxntaP-boBZWOnYY/view, Video showing the comparison of AR overlay accuracy between non-refined optical tracking and refined KF-based optical-inertial tracking.

## Abbreviations

The following abbreviations are used in this manuscript:

AR	Augmented reality
VST	Video see-through
OST	Optical see-through
IMU	Inertial motion unit
GPS	Global Positioning System
KF	Kalman filter
EKF	Extended kalman filter

## Appendix A

The Hausdorff distance $H_{\mathrm{dis}}$ measures how far two subsets of a metric space are from each other (for us the imaging plane $R^{2}$) and it is defined as:

(A1) $$\begin{array}{c} H_{\mathrm{dis}}=\max\left\{\underset{x\in X}{\sup}\underset{y\in Y}{\inf}d\left(x,y\right),\underset{y\in Y}{\sup}\underset{x\in X}{\inf}d\left(x,y\right)\right\} \end{array}$$

where sup is the supremum, inf the infimum, and d(x,y) the denotes the Euclidean distance in $R^{2}$ between points of the two curves.

## References

[1] Grubert J., Itoh Y., Moser K., Swan J.E. (2018). A Survey of Calibration Methods for Optical See-Through Head-Mounted Displays. IEEE Trans. Vis. Comput. Graph..

[2] Cutolo F., Fida B., Cattari N., Ferrari V. (2020). Software Framework for Customized Augmented Reality Headsets in Medicine. IEEE Access.

[3] Cutolo F., Parchi P.D., Ferrari V. Video see through AR head-mounted display for medical procedures. Proceedings of the IEEE International Symposium on Mixed and Augmented Reality (ISMAR).

[4] Zhang Z. (2000). A flexible new technique for camera calibration. IEEE Trans. Pattern Anal. Mach. Intell..

[5] Park J., You S., You S., Neumann U. (1999). Natural Feature Tracking for Extendible Robust Augmented Realities. International Workshop on Augmented Reality: Placing Artificial Objects in Real Scenes (IWAR1998).

[6] Davison A.J., Mayol W.W., Murray D.W. Real-Time Localisation and Mapping with Wearable Active Vision. Proceedings of the Second IEEE and ACM International Symposium on Mixed and Augmented Reality.

[7] Bleser G., Wuest H., Stricker D. Online camera pose estimation in partially known and dynamic scenes. Proceedings of the 2006 IEEE/ACM International Symposium on Mixed and Augmented Reality (ISMAR2006).

[8] Klein G., Murray D. Parallel Tracking and Mapping for Small AR Workspaces. Proceedings of the 2007 6th IEEE and ACM International Symposium on Mixed and Augmented Reality.

[9] Lepetit V., Fua P. (2005). Monocular Model-Based 3D Tracking of Rigid Objects: A Survey. Found. Trends Comput. Graphics Vis..

[10] Kato H., Billinghurst M. Marker Tracking and HMD Calibration for a Video-Based Augmented Reality Conferencing System. Proceedings of the 2nd IEEE and ACM International Workshop on Augmented Reality.

[11] Vávra P., Roman J., Zonča P., P.Ihnát, Němec M., Kumar J., Habib N., El-Gendi A. (2017). Recent Development of Augmented Reality in Surgery: A Review. J. Healthc. Eng..

[12] Cutolo F. (2019). Letter to the Editor on “Augmented Reality Based Navigation for Computer Assisted Hip Resurfacing: A Proof of Concept Study”. Ann. Biomed. Eng..

[13] Ferrari V., Cutolo F., Calabrò E.M., Ferrari M. [Poster] HMD Video see though AR with unfixed cameras vergence. Proceedings of the IEEE International Symposium on Mixed and Augmented Reality (ISMAR).

[14] Kytö M., Nuutinen M., Oittinen P. Method for measuring stereo camera depth accuracy based on stereoscopic vision. Proceedings of the Three-Dimensional Imaging, Interaction, and Measurement.

[15] Cutolo F., Ferrari V. (2018). The Role of Camera Convergence in Stereoscopic Video See-through Augmented Reality Displays. Int. J. Adv. Comput. Sci. Appl..

[16] Sielhorst T., Sa W., Khamene A., Sauer F., Navab N. Measurement of absolute latency for video see through augmented reality. Proceedings of the 6th IEEE and ACM International Symposium on Mixed and Augmented Reality (ISMAR2007).

[17] Badiali G., Ferrari V., Cutolo F., Freschi C., Caramella D., Bianchi A., Marchetti C. (2014). Augmented reality as an aid in maxillofacial surgery: Validation of a wearable system allowing maxillary repositioning. J. Craniomaxillofac Surg..

[18] Cutolo F., Carbone M., Parchi P.D., Ferrari V., Lisanti M., Ferrari M., De Paolis L.T., Mongelli A. (2016). Application of a New Wearable Augmented Reality Video See-Through Display to Aid Percutaneous Procedures in Spine Surgery. Proceedings of the Augmented Reality, Virtual Reality, and Computer Graphics (AVR2016).

[19] Cutolo F., Meola A., Carbone M., Sinceri S., Cagnazzo F., Denaro E., Esposito N., Ferrari M., Ferrari V. (2017). A new head-mounted display-based augmented reality system in neurosurgical oncology: A study on phantom. Comput. Assist. Surg..

[20] Gao X.S., Hou X.R., Tang J., Cheng H.F. (2003). Complete solution classification for the perspective-three-point problem. IEEE Trans. Pattern Anal. Mach. Intell..

[21] Aron M., Simon G., Berger M.O. (2007). Use of inertial sensors to support video tracking. Comput. Animat. Virt. Worlds.

[22] State A., Hirota G., Chen D.T., Garrett W.F., Livingston M.A. (1996). Superior Augmented Reality Registration by Integrating Landmark Tracking and Magnetic Tracking. Proceedings of the 23rd Annual Conference on Computer Graphics and Interactive Techniques.

[23] Yokokohji Y., Sugawara Y., Yoshikawa T. Accurate image overlay on video see-through HMDs using vision and accelerometers. Proceedings of the IEEE Virtual Reality 2000 (Cat. No.00CB37048).

[24] Satoh K., Anabuki M., Yamamoto H., Tamura H. A hybrid registration method for outdoor augmented reality. Proceedings of the IEEE and ACM International Symposium on Augmented Reality.

[25] Jiang B., Neumann U., Suya Y. A robust hybrid tracking system for outdoor augmented reality. Proceedings of the IEEE Virtual Reality 2004.

[26] Klein G., Drummond T. Tightly Integrated Sensor Fusion for Robust Visual Tracking. Proceedings of the British Machine Vision Conference (BMVC’02).

[27] Bleser G., Stricker D. Advanced tracking through efficient image processing and visual-inertial sensor fusion. Proceedings of the 2008 IEEE Virtual Reality Conference.

[28] Ercan A.O., Erdem A.T. On sensor fusion for head tracking in augmented reality applications. Proceedings of the 2011 American Control Conference.

[29] Oskiper T., Samarasekera S., Kumar R. Multi-sensor navigation algorithm using monocular camera, IMU and GPS for large scale augmented reality. Proceedings of the 2012 IEEE International Symposium on Mixed and Augmented Reality (ISMAR).

[30] Menozzi A., Clipp B., Wenger E., Heinly J., Dunn E., Towles H., Frahm J., Welch G. Development of vision-aided navigation for a wearable outdoor augmented reality system. Proceedings of the 2014 IEEE/ION Position, Location and Navigation Symposium - PLANS 2014.

[31] He H., Li Y., Guan Y., Tan J. (2015). Wearable Ego-Motion Tracking for Blind Navigation in Indoor Environments. IEEE Trans. Autom. Sci. Eng..

[32] Qian K., Zhao W., Ma Z., Ma J., Ma X., Yu H. (2018). Wearable-Assisted Localization and Inspection Guidance System Using Egocentric Stereo Cameras. IEEE Sens. J..

[33] Trivisio, Lux Prototyping. https://www.trivisio.com/.

[34] Fontana U., Cutolo F., Cattari N., Ferrari V. Closed - Loop Calibration for Optical See-Through Near Eye Display with Infinity Focus. Proceedings of the 2018 IEEE International Symposium on Mixed and Augmented Reality Adjunct (ISMAR-Adjunct 2018).

[35] Cutolo F., Fontana U., Ferrari V. (2018). Perspective Preserving Solution for Quasi-Orthoscopic Video See-Through HMDs. Technologies.

[36] Cattari N., Cutolo F., D’amato R., Fontana U., Ferrari V. (2019). Toed-in vs Parallel Displays in Video See-Through Head-Mounted Displays for Close-Up View. IEEE Access.

[37] VTK, The Visualization Toolkit. https://vtk.org/.

[38] OpenCV, Open Source Computer Vision Library. https://opencv.org/.

[39] Lee J., Park J., Lee G., Howard D., Kang J.J., Ślęzak D. (2012). Reducing Gross Errors of Perspective 3-point Pose Computation. Proceedings of the Convergence and Hybrid Information Technology.

[40] Arun K.S., Huang T.S., Blostein S.D. (1987). Least-Squares Fitting of Two 3-D Point Sets. IEEE Trans. Pattern Anal. Mach. Intell..

[41] Cutolo F., Freschi C., Mascioli S., Parchi P.D., Ferrari M., Ferrari V. (2016). Robust and Accurate Algorithm for Wearable Stereoscopic Augmented Reality with Three Indistinguishable Markers. Electronics.

[42] Chang C., Chatterjee S. Quantization error analysis in stereo vision. Proceedings of the Conference Record of the Twenty-Sixth Asilomar Conference on Signals, Systems Computers.

[43] Luhmann T., Fraser C., Maas H.G. (2016). Sensor modelling and camera calibration for close-range photogrammetry. ISPRS J. Photogramm. Remote Sens..

[44] Wu Y., Hu Z. (2006). PnP Problem Revisited. J. Math. Imaging Vis..

[45] Ting W., Yuecao W., Chen Y. Some Discussion on the Conditions of the Unique Solution of P3P Problem. Proceedings of the 2006 International Conference on Mechatronics and Automation.

[46] Faugère J.C., Moroz G., Rouillier F., El Din M.S. Classification of the Perspective-Three-Point Problem, Discriminant Variety and Real Solving Polynomial Systems of Inequalities. Proceedings of the Twenty-First International Symposium on Symbolic and Algebraic Computation.

[47] Lepetit V., Moreno-Noguer F., Fua P. (2008). EPnP: An Accurate O(n) Solution to the PnP Problem. Int. J. Comput. Vis..

[48] Garro V., Crosilla F., Fusiello A. Solving the PnP Problem with Anisotropic Orthogonal Procrustes Analysis. Proceedings of the 2012 Second International Conference on 3D Imaging, Modeling, Processing, Visualization Transmission.

[49] Lu C., Hager G.D., Mjolsness E. (2000). Fast and globally convergent pose estimation from video images. IEEE Trans. Pattern Anal. Mach. Intell..

[50] Diotte B., Fallavollita P., Wang L., Weidert S., Euler E., Thaller P., Navab N. (2015). Multi-Modal Intra-Operative Navigation During Distal Locking of Intramedullary Nails. IEEE Trans. Med. Imaging.

[51] He C., Kazanzides P., Sen H.T., Kim S., Liu Y. (2015). An Inertial and Optical Sensor Fusion Approach for Six Degree-of-Freedom Pose Estimation. Sensors.

[52] Park F.C., Martin B.J. (1994). Robot sensor calibration: solving AX=XB on the Euclidean group. IEEE Trans. Robot..

[53] Markley F.L., Cheng Y., Crassidis J.L., Oshman Y. (2007). Averaging Quaternions. J. Guid. Control Dyn..

[54] Wertz J.R. (1978). Three-Axis Attitude Determination Methods. Spacecraft Attitude Determination and Control.

[55] Mamone V., Viglialoro R.M., Cutolo F., Cavallo F., Guadagni S., Ferrari V., De Paolis L.T., Bourdot P., Mongelli A. (2017). Robust Laparoscopic Instruments Tracking Using Colored Strips. Augmented Reality, Virtual Reality, and Computer Graphics. Augmented Reality, Virtual Reality, and Computer Graphics (AVR2017).

[56] Chan T.F., Sandberg B., Vese L.A. (2000). Active Contours without Edges for Vector-Valued Images. J. Vis. Commun. Image Represent..

[57] Lam L., Lee S., Suen C.Y. (1992). Thinning methodologies-a comprehensive survey. IEEE Trans. Pattern Anal. Mach. Intell..

[58] Lu X.X. (2018). A Review of Solutions for Perspective-n-Point Problem in Camera Pose Estimation. J. Phys. Conf..

[59] Okuma K., Taleghani A., de Freitas N., Little J.J., Lowe D.G., Pajdla T., Matas J. (2004). A Boosted Particle Filter: Multitarget Detection and Tracking. Computer Vision - ECCV 2004.

[60] Chang C., Ansari R. (2005). Kernel particle filter for visual tracking. IEEE Signal Process. Lett..

[61] Kosmopoulos D.I., Doulamis N.D., Voulodimos A.S. (2012). Bayesian filter based behavior recognition in workflows allowing for user feedback. Comput. Vis. Image Underst..

